# A Decade of Waiting: Experiences of Women Living With Vulvar Crohn’s Disease and Interactions With Healthcare Professionals Related to Their Sexual Well-Being: A Qualitative Study

**DOI:** 10.1093/crocol/otad025

**Published:** 2023-05-12

**Authors:** Simona Fourie, Debra Jackson, Wladyslawa Czuber-Dochan, Christine Norton

**Affiliations:** Radcliffe Department of Medicine, University of Oxford, Oxford, UK; Florence Nightingale Faculty of Nursing, Midwifery and Palliative Care, King’s College London, London, UK; Faculty of Medicine and Health, University of Sydney, Sydney, Australia; Florence Nightingale Faculty of Nursing, Midwifery and Palliative Care, King’s College London, London, UK; Florence Nightingale Faculty of Nursing, Midwifery and Palliative Care, King’s College London, London, UK

**Keywords:** IBD, extra-intestinal manifestation, vulvar Crohn’s, patient experience

## Abstract

**Background:**

Vulvar Crohn’s disease is a rare cutaneous manifestation of inflammatory bowel disease and to date, studies have reported on under 300 cases worldwide. The condition has an increased risk of malignancy, and diagnosis is often difficult. Treatment protocols are yet to be developed. This paper aimed to provide the first account of patients’ experience of living with vulvar Crohn’s.

**Methods:**

A previous qualitative study exploring experiences of sexual well-being in inflammatory bowel disease and experiences of discussing sexual well-being with healthcare professionals found 3 participants who self-reported vulvar Crohn’s disease. Data from the whole cohort (*n* = 43) were previously reported. Telephone semi-structured interviews were used for data collection. van Manen’s phenomenology of practice framework informed analysis.

**Results:**

Due to significant differences in experiences, this subgroup of 3 women with vulvar Crohn’s warranted separate attention. The common theme of the group was *A decade of waiting*, describing the major delays experienced in being diagnosed. The symptoms reported appeared to be very severe, and sexual well-being was very negatively affected.

**Conclusions:**

Women with vulvar Crohn’s trust in healthcare professionals was eroded as a result of a decade delay in diagnosis, while the quality of life and relationships suffered.

## Introduction

Although rare^1^ and with the real prevalence uncertain, vulvar Crohn’s disease (VCD) is a cutaneous manifestation of Crohn’s disease (CD) and a variation of perianal disease found in women.^2,3^ These vulvovaginal cutaneous lesions have various clinical presentations: vulvar edema is usually unilateral, ulceration, hypertrophic lesions, and chronic suppuration.^4^ It can be particularly difficult to diagnose in the absence of intestinal involvement (symptoms of active intestinal CD) and often patients require extensive investigation to eliminate sexually transmitted diseases, other skin conditions, and infections. Undergoing assessments for sexually transmitted diseases and infections can have a negative impact on women as it can add stigmatizing feelings associated with such investigations.

The current evidence is based on just under 300 cases worldwide. The largest cohort study to date^5^ reported on 50 patients with VCD, representing 0.25% of the inflammatory bowel disease (IBD) female population reviewed, and found that 80% of them also had perianal fistulas, with vulvar pain as the main symptom. Treatment is not well established^6^ and the disease course is unpredictable, from some genital lesions resolving spontaneously to others remaining unresponsive to medical or surgical treatment. One study reported persistent symptoms for a period of 6 months to 20 years.^3^ A multidisciplinary team (MDT) approach is required and a course of oral antibiotics and surgical debridement is the most common approach.^3^ A variant of genital Crohn’s can also be found in men,^7^ although there is also less literature on this subject and it is beyond the scope of this manuscript. IBD treatment for intestinal symptoms is continued alongside the one for genital lesions. The evidence on the topic is scarce and no qualitative literature was found on the life experiences of women with VCD.

## Materials and Methods

This was part of a larger qualitative study. Three women of the 43 participants self-reported having received a diagnosis of VCD. Although the dataset was included in the initial analysis, the severe clinical and personal experiences of these 3 participants warranted a separate report. Participants were interviewed by telephone, and the interviews lasted around 60 minutes each. The data analysis was guided by van Manen’s framework.^8^ Themes describing and interpreting the essence of their experiences were developed.

### Ethical Considerations

Ethical permission for the study was granted by the University of Oxford ethics committee (R60900/RE001). For the purposes of privacy and anonymity, all participants consented to the publication of anonymized excerpts and were given pseudonyms. The direct quotes are verbatim, giving the participants’ pseudonyms and age in italics.

## Results

**Figure 1.**
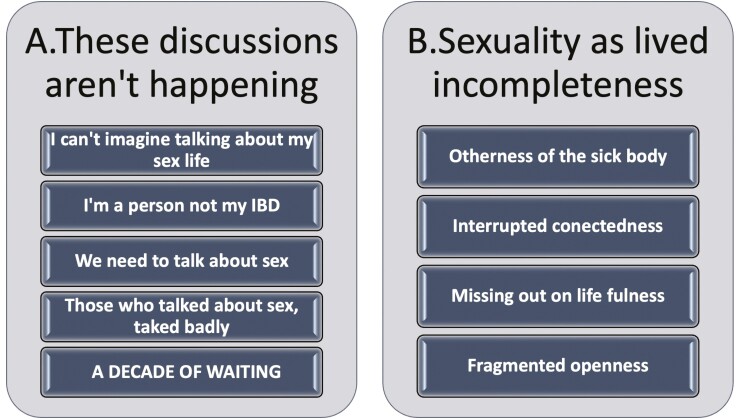
Main themes for (A) lived experiences of discussing sexual well-being with healthcare professionals and (B) lived experiences of intimacy and sexuality in the context of inflammatory bowel disease.

The findings were incidental and were reported by 3 participants from a total of 43 taking part in a previously reported study exploring experiences of intimacy and sexuality in the context of IBD, and their experiences of discussing their concerns with healthcare professionals (HCPs).^9^ The 3 women living with VCD were all over 30 and had undergone previous surgical interventions related to their IBD, also had active perianal disease, other extra-intestinal IBD manifestations such as joint and eye manifestations, and displayed symptoms of a severe course of disease. The themes are summarized in Figure 1 and were described in detail in previous publications.^9,10^

However, their experiences of discussing sexuality concerns with HCPs were more negative than other people we interviewed with IBD. Under the overarching theme defining their experiences of discussing this with HCPs, *t-These discussions aren’t happening*, a commonality in their experience was described as *a decade of waiting*, which was a separate theme for this group. The most significant aspect of the experience in discussing sexuality with HCPs was reflected in this shared theme, as they all struggled to get their concerns across, to be believed, and ultimately, to receive the diagnosis and appropriate care for VCD. Here are those additional aspects of the experiences that were specific to this group*: Bodily changes in VCD, Impact of VCD on romantic relationships, and i-Interactions with HCPs*.

### Bodily changes in VCD

Those who self-reported VCD described the changes in their body image in depth, particularly the vulvar changes they have experienced, which were not encountered in any other women with perianal disease interviewed. All 3 women had major negative feelings regarding their bodies. Martha described how IBD affected her body immediately after starting the interview:

*With my vulva …. I have a very engorged, enlarged vulva …its very swollen and lots of fissuring. And fissuring it goes purple in colour, so my skin goes purple, discoloured, it gets very dry, it peels…. lots of oedema, so water comes out of the skin and, because I’ve got a rectovaginal fistula, I have some discharge and I often get infections* (Martha, 38).

Another participant described in detail what had happened to her body since she was diagnosed with IBD, but particularly since VCD diagnosis:

*So, at the moment I’ve got a stoma, I’ve had that for near 15 years. My Crohn’s in my stomach and stuff its fine, it’s not too bad, but I now have vulvar Crohn’s. I had to have stoma surgery because I’ve got a fistula on the outside of my rectum so I had that removed, the rectum, the whole lot was taken away when I was 25, and the wound for the rectum wouldn’t heal and I ended up spending 9 months in hospital…. That kind of healed up after 9 months of intensive treatment and the next thing is, I started getting sores on my private area. And obviously, with the Crohn’s skin and the discharge, my life has been miserable. I’ve had lots of surgery on my labia, plastic surgery to remove lesions because they are like really deep ulcers and they end up going black and I’ve had to have my labia removed. My vulva looks awful now it’s not like normal, it was just nice and tidy* (Catriona, 43).

The third participant also described how VCD affected her body, especially as she had additional perianal and rectovaginal fistulating disease:

*Currently, I have got a J pouch, I’ve also got four active fistulas and an active recto-vaginal fistula. I’ve just went in for surgery cos I’ve got vaginal Crohn’s, because I’ve got a hole in the side of my vagina* (Sara, 46).

### Romantic elationships and VCD

Their romantic relationships were affected to a major degree. This was possibly due to the lesions directly affecting genital areas, which added to the sexual dysfunction already experienced as a result of intestinal IBD. Two of the three women reported broken-down relationships as a consequence of the IBD, 1-one chose to remain childless, and another was single at the time of the interview. One expressed her fears of her relationship breaking down, as she believed VCD could be the cause for this:

*I’ve had four boyfriends since I’ve had my stoma and no, they’ve all been fine with it, but I think the Crohn’s vulva thing eventually causes [sexual] issues* (Catriona, 43).

Another mentioned that she would choose to be celibate if she lost her current partner:

*I don’t think anyone would be able to encourage me to have sex if my husband dies. I will most definitely stay celibate and on my own because there is no support there and no care. I feel sorry for ladies that are in my situation that don’t have a partner because I imagine they’re leading a very, solitary, alone life* (Sara, 46).

Some severe changes to the vulva as a result of VCD affected partners just as much according to 1 participant:

*We ended up having a bit of an argument and then I showed him one of the photographs and he started crying, and just got really upset because he said he didn’t know it was that bad. You know, it’s hard because I want to have a normal relationship and I can’t, and he’s very good, he’s very understanding and everything, but you know, for my mental health, it really bothers me* (Catriona, 43).

All 3 participants shared their difficulties of having intercourse with VCD, and they revealed distressing experiences:

*I don’t tell my partners that it’s so painful. I don’t tell them, you just get through it, you grin and bear it, you grit your teeth and you smile, and make the right noises. But it can be horrifically painful, really painful and relationships suffer, relationships break down* (Martha, 38).

### Interactions with Healthcare Professionals

From the experiences of this subgroup, it became apparent that women struggle to get the diagnosis of VCD, and they suffered in silence for long time before getting the care they needed. Interactions with their HCPs were strained over the years, as they felt they were not listened to, or HCPs did not recognize the symptoms, and subsequently, some decided to ask for a change in their clinical team. For the 3 participants, the issue of not having their needs met was felt more acutely than other participants in the larger study:

*I was explaining a new hole in my labia to the registrar and he popped back into the consultant, who I could hear in the other room, and he said “Who is it?” and he said “Oh, its [participant name]” and the consultant said “Oh, let sleeping dogs lie” and the registrar came back in the room and said to me: “Oh no, we’re not going to do you an MRI, we’ll just leave it”* (Sara, 46).

The response in addressing her concerns by her clinical team had damaged the patient/clinician relationship to the point of accessing a new clinician who recognized the importance of the issue for participant as well as recognized a condition difficult to treat:

*And the letter I’ve got from her [Consultant] saying today is “Your labia shows that it is swollen particularly on the left side and is symptomatic for Crohn’s”. So, I just feel quite like a lot of weight has lifted that someone is believing me that I’ve got this thing in the side of my vagina* (Sara, 46).

Apart from the difficulties they had in conveying to HCPs the distressing symptoms, all 3 participants struggled with getting a diagnosis:

*It took me years to get my vulvar Crohn’s diagnosed properly. It’s a running joke now because you go to someone and say I’m having problems having intimacy and a sexual relationship, and the first thing someone does, is tell you to get tested for an STD, or that you have cancer, rather than saying this person has IBD we need to sort out this problem. And then when I did get sent to a gynaecologist, they were like we don’t think that we can do anything about this. And the message was, maybe you should give up on that part of your life, maybe that isn’t in your future to have a sex life* (Martha, 38).

Participants perceived that HCPs were reluctant to acknowledge symptoms related to anatomical parts of the sexual/ reproductive system:

*I’ve talked about my [vaginal] pain on so many occasions with an IBD specialist, with GP’s, with my surgical team, and I’ve never ever once had someone take it seriously. I’ve never seen someone be curious about it, and I’ve never seen a doctor that has helped me get resolution* (Martha, 38).

It appeared that the unsatisfactory response given by HCPs to the distressed participants had eroded the patient/HCP relationship, and issues of lack of trust emerged. It is unclear what was the cause for participants to feel their symptoms were not taken seriously, and some felt that current guidelines for specialist referral aimed mainly at cancer detection were not helping with their diagnosis:

*Maybe just the amount of times I’ve been to clinics and its only when I start bleeding in that area and its suddenly a cancer issue, and therefore they are mandated to do something about me. That is how I got my gynaecological referral, but me going for two or three years, me not being able to get pregnant, me, you know all these problems I’d experienced, they somehow meant nothing until they were mandated by guidelines to push for me to go on a cancer referral* (Martha, 38).

Lack of HCP awareness or knowledge of the condition was also evident from the participants’ reports:

*So again, they had never seen vulvar Crohn’s; I had all sorts of doctors involved, like sexual health doctors, and nobody could figure it out. And then in the end it was a nurse who worked it out* (Catriona, 43).

By contrast, HCPs who recognized the impact of the VCD symptoms were appreciated by participants as they felt validated:

*Actually, it’s the colorectal doctor who’s been amazing and said to me, “Jesus, there’s no way you could be intimate like that, and you’re so sore. I hope you’ve got someone looking after you (*Catriona, 43).

However, the absence of guidance regarding a multidisciplinary approach for VCD treatment left one of the participants feeling that her needs would continue to remain unmet for some time:

*The gynae say it’s not a gynae problem, and it’s my Crohn’s. And I go to my Crohn’s doctors, and they haven’t got a clue because it’s all to do with my gynae bits. So, I’m being passed from pillar to post, you know. Or dermatology. So, I’m just getting passed around and it is getting very hard* (Catriona, 43).

Those who searched for peer support when the information from HCPs was not found, raised their concerns that there are others that may be still undiagnosed:

*I’m seeing that on my Crohn’s groups like I’m on a bit of a weird one. I watch it, I don’t really comment much, though I have seen two ladies who are having the same problems and I’ve said to them it’s your Crohn’s, and demand that you have a test because it does a lot of damage very quickly* (Catriona, 43).

Furthermore, the need for psychological support was perceived as being potentially more acute within this subgroup than in the others with IBD but without VCD:

*‘I think yeah, we need to speak about it, and even offering counselling or something… At the moment I am told I will have to live with this for the rest of my life, and how can I do that? Nothing like that, there’s just antidepressants because I’ve got so much going on and I’m depressed, and I’m kind of feeling a bit desperate that I’m not getting any answers’* (Catriona, 43).

## Discussion

There are no other qualitative studies to our knowledge that have reported on the experience of living with VCD. In addition to the previously reported results on the experience of people living with IBD talking with HCPs, there were some unique and striking findings related to living with VCD. The 3 participants described what is it like to be intimate when you live with VCD, and their interactions with HCPs that were believed to lead to a long delay in the diagnosis of VCD. The extent of the physical problems described by these 3 participants was hard to anticipate as although the information from the literature regarding VCD had been graphic and objective, it does not enable constructing a picture of the extent of the trauma that it could cause.

The evidence in the literature on VCD is based on under 300 participants worldwide,^1,11–14^ hence the rich data from the 3 women taking part in this study offered valuable information to be used in practice, giving HCPs a better understanding of the severity of the symptoms and the negative impact on their lives.

The greater impact of VCD on intimacy and sexuality was suggested by the unexpected proportion of respondents to the study (3/43) who self-reported VCD. The rate of VCD respondents may also be of significance in terms of the potential of underreporting symptoms of the condition. The nature of the symptoms and the potential for invasive physical examination could be a major deterrent for reporting such symptoms for certain groups, particularly younger women, or of different cultural backgrounds. There is limited data on vaginal complaints of those with VCD, but a case series of 50 found that 22% of the women with VCD reported vaginal symptoms. It was possible that other associated symptoms present at the time minimized the vaginal manifestations.^13^ One case study reported that prior to a VCD diagnosis, the patient had well-controlled CD, and was in remission, but had ocular extra manifestations of IBD.^2^

Difficulties in getting a VCD diagnosis reported by these women in our study are consistent with the evidence found in the literature.^4,15^ This could be due to the complexity of the VCD, also known as the “great imitator”; VCD can take various forms and similarities with other genital conditions and requires a multidisciplinary approach.^14^ Also, in some cases, VCD precedes the intestinal manifestation,^16,17^ and there are several pediatric cases reporting the cutaneous form of the first manifestation of CD,^18–20^ indicating the need for timely diagnosis and treatment.

There is confirmation that those with VCD have an increased risk of developing malignancies, especially those presenting with anal fistulas as well.^12^ A study on a cohort of 13 women with VCD found an increased level of vulvar malignancy in their cohort, as 23% of them developed vulvar dysplasia and/or malignancy.^15^ Considering the increased risk of malignancy associated with VCD, better algorithms for early diagnosis should be introduced, especially as pediatric VCD cases have been reported.^1,11^ This is particularly important as there seemed to be significant delays in the diagnosis of VCD that added distress and led to poorer outcomes, as some women would need excision surgery if not treated in time.^4^

Also, the multidisciplinary approach was found by participants to be largely nonexistent, although a multidisciplinary approach is required to achieve disease control.^21^ An holistic approach to care is imperative, as those living with VCD are more likely to experience negative mental health comorbidities as a high number of them undergo surgical procedures for CD,^22^ and have a greater potential to report a diminished quality of life due to implications for their sexual well-being, as reported by participants in our study.

Better detection strategies within IBD services and early MDT discussion should improve the health outcomes for this group. A tool for early detection of VCD could be developed, although a questionnaire could be also effective. An existing IBD-related disability self-assessment tool (IBD Disk) could be potentially used to identify and discuss issues specific to each patient,^23^ and could be helpful in detecting (by discussion with patients) any VCD symptoms if the sexuality component of the tool score is low. Finally, as qualitative research in IBD is now getting increased recognition for addressing the gap between clinicians’ and patients’ priorities, as it covers more holistically aspects of life valued by patients,^24^ we hope that this paper would help HCPs understand life with VCD and prevent delays in diagnosis.

### Limitations

The results were drawn from a very small sample and it is an interpretation of the data; therefore, there is no claim that the results are generalizable. Participants were all self-selected by opting to volunteer for the study, from the United Kingdom and accessed the National Health Service. They were all Caucasian; therefore, experiences in other ethnic groups are unknown. The impact of cultural stigma could be a factor contributing to underreporting of symptoms and could have also contributed to the underrepresentation of a diverse population in this research project.

## Conclusions

There are significant delays in getting a diagnosis of VCD, even in those with diagnosed CD and vaginal symptoms. Sometimes this erodes the patient’s trust in HCPs. The participants in our study experienced a decade of delays for their reported symptoms and concerns to conclude in a formal diagnosis. Their symptoms appear more debilitating than other forms of perianal disease found in IBD. As a result, their quality of life had been significantly reduced, affecting their sexual well-being and relationships with their partners.

### Implications for Practice

Improving communication between the MDT members could lead to early detection of VCD, increased patient satisfaction with service delivery, alongside improved health-related outcomes and reduced risk of malignancy. Specialists like gastroenterologists, gynecologists, dermatologists, psychologists, and sexual health counselors should be part of an MDT involved in the care of vulvar Crohn’s patients, and some of them should be specialized in IBD, similarly to how some gastroenterologists specialize in IBD, some gynecologists are specialists in vulvar diseases. Actively asking patients about intimacy issues could potentially help earlier detection of vaginal symptoms related to VCD. A culturally competent approach is needed for detection and effective cross-cultural communication reduces health disparities. Psychological assessment and support should be integrated in the care of all those with VCD as they have significant additional symptoms to the existing burden of IBD. More research is needed to try and establish the extent of VCD, as it is possible that it is more prevalent than current evidence suggests. The impact of VCD on patients’ life suggests the need for HCPs to be more mindful of the effects of illness and the plethora of new symptoms, fears, and concerns that it brings with it.

## Supplementary Data

The data underlying this article cannot be shared publicly for the privacy of individuals that participated in the study. The data will be shared on reasonable request to the corresponding author.

## Conference Presentation

Part of this work was presented at the 16^th^ Congress of European Crohn’s and Colitis Organisation, July 2021, as a poster. This is also a subset of data from an unpublished PhD thesis from 2022.
